# Induction kinetics of the *Staphylococcus aureus *cell wall stress stimulon in response to different cell wall active antibiotics

**DOI:** 10.1186/1471-2180-11-16

**Published:** 2011-01-20

**Authors:** Vanina Dengler, Patricia Stutzmann Meier, Ronald Heusser, Brigitte Berger-Bächi, Nadine McCallum

**Affiliations:** 1 Institute of Medical Microbiology, University of Zurich, Gloriastr. 32, 8006 Zurich, Switzerland

## Abstract

**Background:**

*Staphylococcus aureus *activates a protective cell wall stress stimulon (CWSS) in response to the inhibition of cell wall synthesis or cell envelope damage caused by several structurally and functionally different antibiotics. CWSS induction is coordinated by the VraSR two-component system, which senses an unknown signal triggered by diverse cell wall active agents.

**Results:**

We have constructed a highly sensitive luciferase reporter gene system, using the promoter of *sas016 *(*S. aureus *N315), which detects very subtle differences in expression as well as measuring > 4 log-fold changes in CWSS activity, to compare the concentration dependence of CWSS induction kinetics of antibiotics with different cell envelope targets. We compared the effects of subinhibitory up to suprainhibitory concentrations of fosfomycin, D-cycloserine, tunicamycin, bacitracin, flavomycin, vancomycin, teicoplanin, oxacillin, lysostaphin and daptomycin. Induction kinetics were both strongly antibiotic- and concentration-dependent. Most antibiotics triggered an immediate response with induction beginning within 10 min, except for tunicamycin, D-cycloserine and fosfomycin which showed lags of up to one generation before induction commenced. Induction characteristics, such as the rate of CWSS induction once initiated and maximal induction reached, were strongly antibiotic dependent. We observed a clear correlation between the inhibitory effects of specific antibiotic concentrations on growth and corresponding increases in CWSS induction kinetics. Inactivation of VraR increased susceptibility to the antibiotics tested from 2- to 16-fold, with the exceptions of oxacillin and D-cycloserine, where no differences were detected in the methicillin susceptible *S. aureus *strain background analysed. There was no apparent correlation between the induction capacity of the various antibiotics and the relative importance of the CWSS for the corresponding resistance phenotypes.

**Conclusion:**

CWSS induction profiles were unique for each antibiotic. Differences observed in optimal induction conditions for specific antibiotics should be determined and taken into account when designing and interpreting CWSS induction studies.

## Background

**Figure 1:**
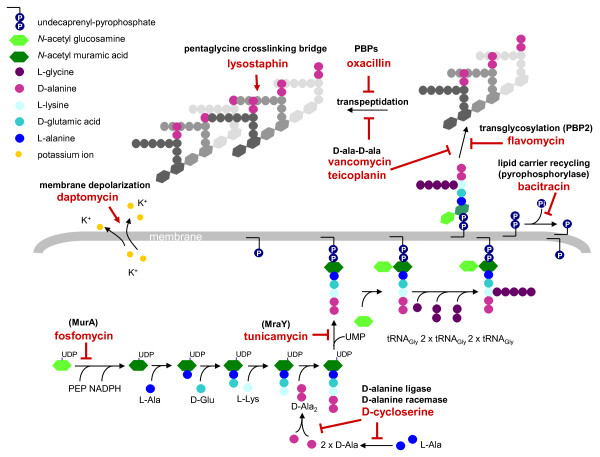
**Schematic representation of the enzymatic steps involved in *S. aureus *cell wall synthesis and the targets of cell wall active antibiotics**. Fosfomycin inhibits the enzyme MurA (UDP-*N*-acetylglucosamine-3-enolpyruvyl transferase) that catalyses the addition of phosphoenolpyruvate (PEP) to UDP-*N*-acetyl-glucosamine (GlcNAc) to form UDP-N-acetyl-muramic acid (UDP-MurNAc) [34]. D-cycloserine prevents the addition of D-alanine to the peptidoglycan precursor by inhibiting D-alanine:D-alanine ligase A and alanine racemase [35]. Tunicamycin is a glycoprotein antibiotic that inhibits the transfer of peptidoglycan precursor (phospho-MurNAc-pentapeptide) to the lipid carrier undecaprenyl pyrophosphate (or C55-isoprenyl pyrophosphate), catalysed by the translocase MraY [36,37]. Sub-lethal concentrations of tunicamycin also inhibit TarO, the first enzyme in the wall teichoic acid pathway [38,39]. Bacitracin forms a metal-dependent complex with the lipid carrier undecaprenyl pyrophosphate, thereby preventing dephosphorylation and the recycling of the lipid carrier required for cell wall synthesis [40,41]. Flavomycin (a moenomycin complex) is a phosphoglycolipid antibiotic that inhibits transglycosylation through binding of the transglycosylase domain of penicillin-binding protein 2 (PBP2) [42]. Glycopeptide antibiotics, such as vancomycin and teicoplanin, inhibit cell wall synthesis by binding the D-ala-D-ala of the lipid II and sterically hindering transglycosylation and transpeptidation. Teicoplanin activity is enhanced through its interaction with the cytoplasmic membrane [43]. ß-lactam antibiotics, such as oxacillin, bind the transpeptidase active domain of penicillin-binding proteins (PBPs) by mimicking the D-ala-D-ala end of the pentapeptide [44]. The mode of action of daptomycin is not fully known, it causes calcium-dependent disruption of membrane function and potassium efflux [45], but was also predicted to directly or indirectly inhibit peptidoglycan systhesis [9]. Lysostaphin is a zinc metalloenzyme that cleaves the pentaglycine crosslinking bridge specific for the cell wall of *S. aureus *[46]. (Adapted from [47]).

*Staphylococcus aureus *is a major cause of both nosocomial and community-acquired infections worldwide. Because staphylococci can adapt rapidly to varying environmental conditions they are quick to develop resistance to virtually all antibiotics and multiple-drug resistance, especially in methicillin-resistant *S. aureus *(MRSA), severely restricts antibiotic therapy options. One of the major targets for antimicrobial agents is the bacterial cell envelope, which is a complex, multi-macromolecular structure that undergoes highly ordered cycles of synthesis and hydrolysis, in order to facilitate cell division while maintaining a protective barrier against environmental stresses. There are several different classes of antibiotics that target specific cell envelope structures or enzymatic steps of cell wall synthesis (Figure 1).

Many antibiotic resistance phenotypes in *S. aureus *are influenced by global regulators that control virulence factors, metabolism and/or stress responses [1]. One of the latter is the VraSR system, which triggers the cell wall stress stimulon (CWSS); a set of genes that is induced in *S. aureus *upon exposure to cell wall active antibiotics, cell wall hydrolysis, or the inhibition of cell wall synthesis, but not by other external stresses, such as temperature, osmotic or pH extremes [1-3]. An unknown signal, responding to cell wall stress, stimulates the intramembrane sensor VraS to activate the response regulator VraR by phosphorylation. When the stress signal is relieved, VraR is subsequently deactivated by VraS-specific dephosphorylation [4].

VraR, depending upon its phosphorylation state, was shown to recognise VraR-responsive promoter sequences and to control the expression of target genes [5]. The phosphorylation kinetics suggested that VraSR signal transduction was likely to respond very rapidly *in vivo *[4]. A general stress signal, rather than the antibiotics themselves, was proposed to initiate CWSS induction [6-8]. This hypothesis is supported by the fact that the CWSS is induced by several different cell wall antibiotics with different targets and/or modes of action as well as by the inhibition of cell wall synthesis resulting from reduction of PBP2 and MurF expression [6,7,9].

Upregulation of the CWSS provides a certain level of resistance/tolerance to most VraSR-inducing agents, although the exact stress response coordinated has not been fully characterised. Core CWSS genes include: *murZ *(MurA isozyme), involved in the early steps of cell wall biosynthesis [10]; *pbp2 *and *sgtB*, involved in transglycosylation; and *fmtA*, a penicillin binding protein with low affinity to β-lactams [3,11,12]. Therefore activation of the CWSS is predicted to enhance cell wall synthesis [2]. This is substantiated by the identification of clinical isolates with point mutations in the *vraSR *operon that lead to increased basal expression of the CWSS in the absence of inducing agents, with the resulting phenotypes including thickened cell walls and increased levels of glycopeptide and ß-lactam resistance [13,14].

The VraSR system of *S. aureus *has been found to be induced by a much wider range of cell wall active antibiotics than the homologous LiaRS systems of *Bacillus subtilis *and *Streptococcus mutans*, which are only induced by lipid II-interacting antibiotics and not by those that inhibit the earlier or later stages of cell wall synthesis [15-18]. However, the sizes and compositions of VraSR regulons reported so far vary quite extensively and appear to be heavily dependent upon the strains and experimental procedures used. Huge variations in levels of CWSS gene induction were found not only to be dependent upon the types of antibiotic used but also on the antibiotic concentrations [2,19,20].

In this study we created a highly sensitive reporter gene construct to indirectly measure the kinetics of VraSR-dependent signal transduction in the presence of antibiotic concentrations ranging from sub- to supra- minimum inhibitory concentrations (MIC), for a selection of antibiotics with different cell envelope targets (Figure 1). This allowed us to compare maximal induction capacities and determine optimal conditions, including concentrations and exposure times, for measuring CWSS induction by different antibiotics.

## Methods

### Bacterial strains and growth conditions

The strains and plasmids used in this study are listed in Table 1. Bacteria were grown at 37°C in Luria Bertani (LB) broth (Difco Laboratories), shaking at 180 rpm with a 1:5 culture to air ratio, or on LB agar plates. All optical density (OD) measurements given were taken at OD _600 nm_. Media were supplemented with the following antibiotics when appropriate: 10 μg/ml tetracycline (Sigma), 10 μg/ml chloramphenicol (Sigma), 100 μg/ml ampicillin (Sigma) or 200 ng/ml anhydrotetracycline (Vetranal). Strains were stored at -80°C in skim milk.

**Table 1:** Strains and plasmids

Strain/plasmid	**Relevant genotype**^***a***^	Reference/source
***S. aureus***		

RN4220	Restriction-negative derivative of NCTC8325-4	[48]

BB255	NCTC8325 derivative, cured of plasmid pI524	[49]

BB255ΔVraR	BB255 containing *vraR *mutation, truncating VraR after the 2^nd ^amino acid	This study

***E. coli***		

DH5α	F^- ^φ80*lac*Z∆M15 ∆(*lac*ZYA-*arg*F)U169 *rec*A1 *end*A1 *hsd*R17(r_k_^-^, m_k_^+^) *pho*A *sup*E44 *thi*-1 *gyr*A96 *rel*A1λ^-^	Invitrogen

**Plasmids**		

pSP-*luc*+	Luciferase fusion plasmid, *ori *ColE1, *bla*, *luc*+; Ap^r^	Promega

pBUS1	*E. coli - S. aureus *shuttle vector, *tetL *; Tc^r^	[31]

pKOR1	*E. coli - S. aureus *shuttle plasmid, for creating markerless deletions; *repF *(ts), *cat*, *attP*, *ccdB*, *ori *ColE1, *bla*, P*xyl*/*tetO, secY570*; Ap^r^, Cm^r^	[25]

pKOR1-VraR::stop	pKOR1 construct containing mutant *vraR *insert with XhoI site and two inframe stop codons inserted between the 2^nd ^and 3^rd ^*vraR *codons.	[26]

p*sas016*_p_-*luc*+	pBUS1 containing the *sas016 *promoter-luciferase reporter gene fusion	[26]

p*tcaA*_p_-*luc*+	pBUS1 containing the *tcaA *promoter-luciferase reporter gene fusion	This study

p*sa0908*_p_-*luc*+	pBUS1 containing the *sa0908 *promoter-luciferase reporter gene fusion	This study

^*a*^Abbreviations: Tc^r^, tetracycline resistance; Ap^r^, ampicillin resistance; Cm^r^, chloramphenicol resistance

### Susceptibility tests

The MICs of antibiotics were determined by Etest (BioMérieux) on LB plates swabbed with an inoculum of 0.5 McFarland and incubated at 37°C for 24 h. The MICs of flavomycin, D-cycloserine, tunicamycin and lysostaphin were determined by microdilution in LB broth, essentially as recommended by the Clinical and Laboratory Standards Institute [21].

### Northern Blots

Northern blots were performed as previously described [22]. Overnight cultures were diluted to OD 0.05 in prewarmed LB containing tetracycline and grown to approximately OD 0.5. Cultures were induced with increasing concentrations of oxacillin and a control culture was grown without antibiotic treatment. Samples were taken after 20 min and 60 min of induction and total RNA was extracted as described by Cheung et al. [23]. RNA samples (7 μg) were separated in a 1.5% agarose-20 mM guanidine thiocyanate gel in 1 × TBE buffer [24]. Digoxigenin (DIG)-labelled probes were amplified using the PCR DIG Probe synthesis kit (Roche) and primer pairs SAS016.for (TCATACGTTCTATGTCTGAT) and SAS016.rev (GATCTATATCGTCTTGTAAT); and luc+ (GGCAATCAAATCATTCCGGATACTG) and luc- (ATCCAGATCCACAACCTTCGCTTC).

### Construction of *vraR *mutant

The pKOR1 system developed by Bae et al. [25] was used to inactivate VraR in BB255, by inserting an XhoI site and two stop codons in-frame into the beginning of the *vraR *coding sequence, truncating VraR after the 2^nd ^amino acid, as previously described [26].

### Luciferase reporter gene fusions

Promoter regions of *sas016 *(SACOL0625)*, tcaA *and *sa0908 *(SACOL1065) were PCR amplified from *S. aureus *strain COL using primer pairs: *sas016*.lucF (AATTAGGTACCTGGATCACGGTGCATACAAC) and *sas016*.lucR (AATTACCATGGCCTATATTACCTCCTTTGC); *tcaA*.lucF (TAATGGTACCAGTATTAGAAGTCATCAATCA) and *tcaA*.lucR (TAAT CCATGGTTTCACCTCAATTCTGTTCCT), and *sa0908*.lucF (AATTAGGTACCATAA TAGTACACACGCATGT) and *sa0908*.lucR (TTAATCCATGGTTGATGCTCCTA TATTAAATT), respectively. PCR products were digested with Asp718 and NcoI and ligated directly upstream of the promoterless luciferase (*luc+*) gene in vector pSP-*luc+ *(Promega). Fragments containing the resulting promoter-*luc+ *translational fusions were then excised with Asp718 and EcoR1 and cloned into the *E. coli - S. aureus *shuttle vector pBUS1 (Table 1). The fusion plasmids p*tcaA*_p_-*luc*+, p*sa0908*_p_-*luc*+ and p*sas016*_p_-*luc+ *(Table 1) were then electroporated into *S. aureus *RN4220 before being transduced, by phage 80α, into *S. aureus *BB255.

### Luciferase assays for quantification of promoter induction

For induction assays, pre-warmed LB broth was inoculated with an overnight culture to an OD of 0.05. Cultures were grown to OD 0.3 - 0.5 and pre-induction samples were collected before the cultures were induced with increasing concentrations of the antibiotics: fosfomycin (disodium salt, Sigma), D-cycloserine (Sigma), bacitracin (from *Bacillus lincheniformis*, Sigma), vancomycin (Vancocin, Eli Lilly), teicoplanin (Hoechst Marion Roussel), oxacillin (InfectoPharm), flavomycin (BC Biochemie GmbH), daptomycin (Cubist Pharmaceuticals), tunicamycin (AG Scientifics) and lysostaphin (ambicin, AMBI). Medium was supplemented with 25 μg/ml ZnCl for bacitracin, 50 μg/ml CaCl_2 _for daptomycin and 25 μg/ml glucose-6-phosphate for fosfomycin experiments. Samples were then collected and the OD measured after 10, 20, 30, 45, 60 and 120 min. For each sample, 1 ml of culture was harvested by centrifugation and the pellets frozen at -20°C. To measure the luciferase activity, pellets were thawed briefly and resuspended in PBS (pH 7.4) to an OD of either 10 or 1, depending on induction levels. Aliquots of the cell suspensions were then mixed with equal aliquots of Luciferase Assay System substrate (Promega) and luminescence was measured for 15 s after a delay of 3 s on a Turner Designs TD-20/20 luminometer (Promega) in relative light units (RLU).

For the determination of colony forming units per millilitre (CFU/ml), 1 ml samples of cultures that had been induced for 120 min with 1xMIC of each antibiotic were harvested by centrifugation. Cell pellets were resuspended in 0.85% NaCl and immediately diluted and plated on sheep-blood agar plates.

## Results and Discussion

### Comparison of CWSS reporter constructs

**Figure 2:**
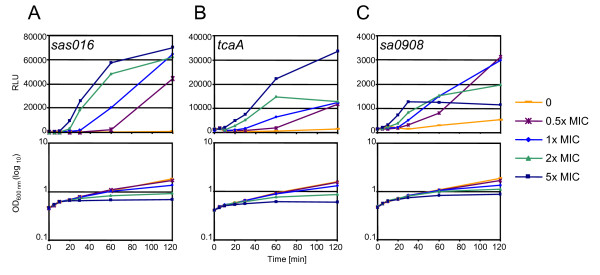
**Induction kinetics of three CWSS promoters in response to varying concentrations of oxacillin**. Luciferase activities and growth curves of strain BB255 containing: A, p*sas016-luc+; *B, p*sa0908-luc+; *and C, p*tcaA-luc+; *after addition of 0, 0.5, 1, 2 or 5-fold the MIC of oxacillin at time point zero.

To quantify CWSS induction and follow its time course upon antibiotic exposure, the promoters of the three representative CWSS genes *sas016*, *sa0908 *and *tcaA*, were fused to the luciferase reporter gene and the resulting plasmids were introduced into antibiotic susceptible strain BB255. *sas016 *encodes a hypothetical protein of unknown function and was the open reading frame (ORF) found to be most strongly up-regulated by cell wall antibiotics in several studies [3,11,20]; *tcaA *encodes a predicted membrane protein that influences glycopeptide resistance and virulence in a nematode model and belongs to the core *S. aureus *CWSS [11,22,27]; and *sa0908 *encodes an envelope protein that influences lytic behaviour in *S. aureus *and is one of a family of three LytR-CpsA-Psr proteins that are all induced by cell wall stress (unpublished results). Increasing concentrations of oxacillin were added to exponentially growing cultures containing reporter plasmids, and the OD and luciferase activities were measured over a two hour period (Figure 2). The three promoters were all induced in a concentration dependent manner, with induction lag times becoming shorter and induction rates steeper as oxacillin concentrations increased. This was mirrored by a corresponding stepwise decrease in growth rates. Induction rates generally began to slow after 60 min, upon the onset of oxacillin induced lysis [28], but again this was concentration dependent with induction rates beginning to decrease earlier in cultures with higher oxacillin concentrations.

Previous findings, using Northern blots to measure oxacillin induction levels of *sas016 *after 30 min, indicated that inhibitory concentrations of oxacillin were required for induction [20]. Figure 2 confirmed that the sub-inhibitory concentration of 0.5x MIC did not noticeably induce promoter activity after 30 min, however, luciferase activity from all three promoters began to increase sharply after 60 min and continued to rise up to the final sampling point of 120 min.

Although all three promoters displayed similar relative concentration- and time-dependent induction kinetics, the *sas016 *promoter produced the highest levels of luciferase activity, resulting in greater fold-changes between samples and making it the most sensitive of the three reporters. Therefore we chose the *sas016 *promoter-luciferase fusion construct as the best indicator to compare induction characteristics of different cell wall active antibiotics.

### Correlation between *sas016 *transcript induction and luciferase activity from p*sas016*_p_*-luc+*

**Figure 3:**
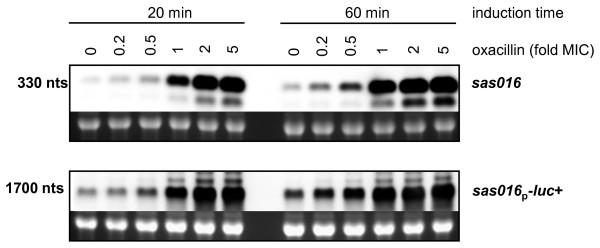
**Northern blot analysis of *sas016 *and *sas016***_**p**_***-luc*+ transcript induction BB255 p*sas016***_**p**_**-*luc*+**. RNA was harvested from cultures after 20 and 60 min of induction with 0, 0.2, 0.5, 1, 2 or 5-fold MIC concentrations of oxacillin. Transcripts hybridising to *sas016 *and *luc+*-specific DIG and their approximate sizes are indicated. Approximate transcript sizes are indicated on the left side of the blots. Ethidium bromide stained 16S rRNA bands are shown below Northern blots as an indication of RNA loading.

To confirm that levels of luciferase activity from p*sas016*_p_-*luc*+ accurately represented levels of *sas016 *gene expression, Northern blots were performed on BB255 p*sas016*_p_-*luc*+ RNA samples extracted from cultures grown using the same conditions and oxacillin concentrations used for luciferase assays. Samples were harvested 20 min and 60 min after antibiotic induction and hybridized with *sas016 *and *luc*+ specific DIG probes (Figure 3). Northern blots showed identical patterns of transcriptional induction for both the chromosomal *sas016 *gene and the luciferase gene under the control of the *sas016 *promoter in p*sas016*_p_-*luc*+. Induction of both transcripts was highly oxacillin-concentration dependent and transcript intensities increased over time becoming stronger after 60 min than after 20 min, correlating very well with concentration-specific induction curves from luciferase assays (Figure 2).

### Antibiotic-dependent induction of the CWSS

**Figure 4:**
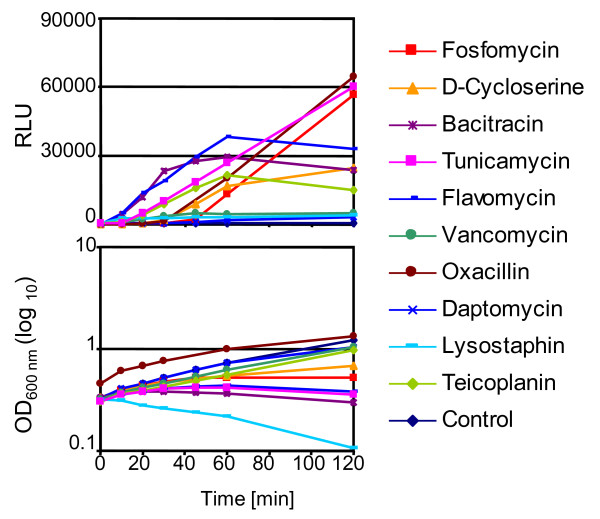
**Antibiotic dependent induction of the cell wall stress stimulon**. The upper graph shows relative light units (RLU) measured upon induction of BB255 p*sas016*_p_-*luc*+ of cultures stressed with 1x MIC of different antibiotics. The corresponding OD values at each sampling point are presented below. The graphs shown are representative results of between two and four induction experiments performed for each antibiotic.

The MIC values of diverse antibiotics chosen for induction experiments were determined for strain BB255 p*sas016*_p_*-luc+ *(Table 2). MIC concentrations were then used in induction experiments to compare the relative inducing capacities of the antibiotics (Figure 4). When adding MIC concentrations of antibiotics to exponentially growing cultures, salient differences in induction kinetics were apparent throughout the two hour sampling period, including the slopes of induction curves and the maximal luciferase activities reached. Large differences were also seen in the response of the culture's ODs over the induction period, which ranged from slight growth retardation, through to halting of growth and decreasing OD readings; reflecting differences in the effectiveness of the antibiotics and the concentrations used, which are likely to impact CWSS induction kinetics. There were no apparent connections between the stages of cell wall synthesis targeted by antibiotics and CWSS induction potential. Oxacillin and fosfomycin, which target completely different enzymatic stages of peptidoglycan synthesis, showed the highest maximal induction levels, with luciferase activity becoming induced relatively late, but then continually increasing over the two hour period. Bacitracin, tunicamycin, D-cycloserine, flavomycin and teicoplanin showed medium levels of induction, although there were large differences in the shapes of their induction curves. Bacitracin and flavomycin initiated induction very rapidly and maximal expression peaked after 60 min. The teicoplanin induction curve was shallower but maximal induction was again reached at 60 min. Vancomycin was a comparably weak inducer at the MIC concentration. Induction by lysostaphin appeared immediately, within the first 10 min, but remained very low. The OD curve for lysostaphin showed significant lysis of the culture, which would account for the overall low levels of luciferase measured. Induction therefore seems to be more strongly influenced by the specific activities of the different antibiotics used, rather than their targets.

**Table 2:** MIC values and summary of induction kinetics characteristics of different antibiotics

Antibiotic	**MIC**^*a*^	**Fold MIC decrease in BB255ΔVraR**^*b*^	**Lag before induction**^*c*^	**Maximum induction**^*d*^	**Time point of maximum induction**^*e*^	**Concentration dependence**^*f*^	**OD/CFU/ml as % of control**^*g*^
Fosfomycin	0.5	2x	30	high	120	high (29.5)	47/10

D-Cycloserine	12	none	10	medium	60	high (25.5)	56/36

Bacitracin	32	10x	none	medium	60	low (1.5)	26/9

Tunicamycin	8	4x	10	high	120	medium (3.0)	38/9

Flavomycin	4	16x	none	high	60	low (1.6)	41/25

Vancomycin	1.3	2x	none	low	120	medium (12.6)	100/100

Oxacillin	0.2	none	none	high	120	high (19.1)	74/20

Daptomycin	0.25	2x	none	low	120	medium (14.1)	85/75

Lysostaphin	0.065	2x	none	low	10	medium (11.3)	11/6

Teicoplanin	0.5	10x	none	medium	60	medium (7.5)	91/83

^*a*^Determined in μg/ml for BB255 p*sas016*-*luc*+. ^*b*^Difference in MIC values of BB255/BB255ΔVraR. ^*c*^Earliest time point at which induction was detected (min). ^*d*^Induction levels were scored as: high (> 40'000 RLU); medium (>10'000 - < 40'000); low (< 10'000). ^*e*^Time taken for maximum induction to be reached after antibiotic addition (min). ^*f*^The ratio of maximal induction levels measured at 5x MIC/0.2x MIC, scored as: high (> 15); medium (>2 - < 15); low (< 2). ^*g *^OD and CFU/ml values after treatment with antibiotics (1x MIC) for 120 min, expressed as a percentage of the values from untreated cell.

### Concentration-dependent CWSS induction kinetics

**Figure 5:**
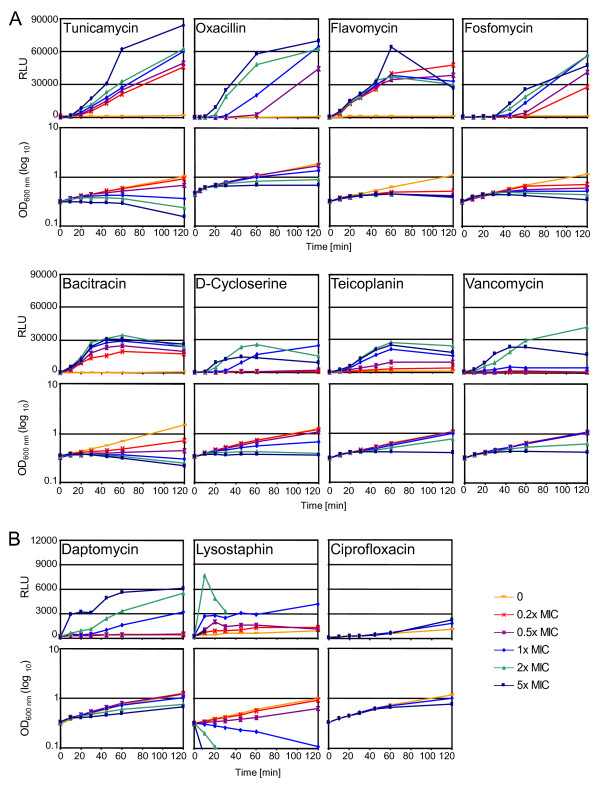
**Concentration-dependent cell wall stress stimulon induction kinetics of different cell wall active antibiotics**. Graphs show relative light units (RLU) measured upon induction of BB255 p*sas016*_p_-*luc*+ with five different antibiotic concentrations and the corresponding OD values at each sampling point. The graphs shown are representative results of between two and four induction experiments performed for each antibioti**c**. A, concentration-dependent induction kinetics of antibiotics scored as high- or medium-level inducers. B, concentration-dependent induction kinetics of antibiotics scored as low-level inducers and the fluoroquinolone antibiotic ciprofloxacin.

Large differences were observed in the CWSS induction kinetics of antibiotics when used at MIC levels, however, these concentrations may not have represented the optimal induction conditions for all of the antibiotics. Therefore, induction assays were performed as above, but using five different antibiotic concentrations ranging from sub- up to supra-inhibitory (Figure 5). Additionally, ciprofloxacin, a flouroquinolone antibiotic that does not target the cell envelope was included as a control at concentrations of 2x and 5x the MIC (MIC = 0.2 μg/ml).

Tunicamycin, flavomycin, oxacillin and fosfomycin triggered the highest maximal induction levels (RLU > 40'000) (Figure 5A, Table 2). Bacitracin, D-cycloserine, teicoplanin, and vancomycin showed medium levels of induction (RLU > 10'000 - < 40'000), while daptomycin and lysostaphin were the weakest inducers (RLU < 10'000) (Figure 5, Table 2). Daptomycin is known to target the bacterial cell membrane, causing membrane depolarization and potassium efflux (Figure 1), however because of its ability to trigger the CWSS it is also thought to directly or indirectly interfere with peptidoglycan synthesis [9], although the mechanism by which this occurs is unknown.

Ciprofloxacin treated cells showed no luciferase induction after 60 min and although levels were up to 2-fold higher than in untreated cells after two hours, no further increases in expression were detected, even after four hours when the OD started to decrease in response to the ciprofloxacin treatment. Therefore marginal increases were unlikely to be caused by ciprofloxacin-specific induction of the CWSS as even the lowest inducers, lysostaphin and daptomycin, stimulated 18-fold and 14-fold induction, respectively.

Shapes of the induction curves were different for all of the antibiotics tested. Most of the antibiotics triggered immediate induction of the CWSS, with lysostaphin producing the strongest and most rapid response within the first 10 min, followed by flavomycin, bacitracin, daptomycin, vancomycin, teicoplanin and oxacillin. Contrarily, fosfomycin and D-cycloserine showed a lag phase of induction for all concentrations of approximately 30 min and 10 min, respectively, before any induction could be detected. Tunicamycin also showed a 10 min lag phase for all concentrations except 5x MIC, for which a slight 3-fold induction could be measured at the 10 min sampling point. Fosfomycin, D-cycloserine and tunicamycin act on early steps of peptidoglycan synthesis (Figure 1), which could be linked to the lags in CWSS induction. Balibar et al. also detected a lag phase of CWSS induction when *S. aureus *was treated with the UPP synthesis inhibitor hymeglusin [29].

Concentration-dependence was categorized based on the spread of the induction curves, so that antibiotics with large distances between the curves for different concentrations were scored as being highly concentration-dependent; while those in which the majority of curves clustered closely together were scored as having low dependence. The concentration-dependency of induction was also evaluated by determining the ratio of the induction measured at 5x MIC over that at 0.2x MIC (Table 2). Accordingly, fosfomycin, D-cycloserine, oxacillin, tunicamycin, vancomycin, daptomycin and lysostaphin showed relatively high concentration-dependency (ratio >2). Some of these antibiotics such as fosfomycin, oxacillin and daptomycin had quite evenly spread curves that generally increased incrementally as concentrations became higher. Whereas for vancomycin, there was a gap between the supra-MIC curves which both showed relatively high induction, and all of the sub-MIC curves that exhibited very little induction. In different experiments curves corresponding to the vancomycin MIC vacillated between showing either mid-level induction or clustering with the sub-MIC curves, indicating that the MIC of vancomycin was very close to the threshold concentration required for CWSS induction. Flavomycin and bacitracin induction curves also increased incrementally as concentrations increased, but the gaps between the curves were much smaller than for most of the other antibiotics (ratio < 2).

Previous studies have reported contradictory results regarding the induction of the CWSS by lysostaphin. Some studies detected no induction of the CWSS by lysostaphin [19,30], while Rossi et al. detected a slight induction of the CWSS gene *mrsR *upon lysostaphin treatment [31]. Possible reasons for these discrepancies are likely to be linked to experimental variations in the strains, lysostaphin concentrations and induction times used, or the sensitivity of induction detection methods. In this study, lysostaphin induction could only be detected under very specific experimental conditions (Figure 5B).

The influences of antibiotic concentrations on CWSS induction kinetics generally correlated closely with the impacts of the corresponding concentrations on the OD of the cultures (Figure 5). For example, the incremental increases in oxacillin induction curves closely mirrored corresponding decreases in culture OD curves. For flavomycin, all of the concentrations used induced luciferase activity to similar levels and all growth curves were correspondingly inhibited to similar extents. All experiments showed a definite correlation, albeit to different extents, between levels of growth arrest in the cultures and corresponding levels of CWSS induction. This trend is not always proportional, however, as bacitracin and tunicamycin OD curves showed a large degree of spread whereas induction curves were more closely clustered.

To compare how decreases in OD correlated with cell viability, CFU/ml were measured after treatment with 1x MIC of each antibiotic for two hours. The percentage decrease in CFU/ml generally corresponded well with the percentage decrease in OD (Table 2).

### Impact of VraR inactivation on resistance to the cell wall antibiotics tested

Deletion of the *vraSR *operon is known to decrease resistance levels to most of its inducing antibiotics [2,6,9,32]. However, the reported effects on different resistance phenotypes varied greatly, with some MICs unaffected while others were decreased up to 40-fold; indicating that induction of the CWSS is more essential for protecting *S. aureus *against some antibiotics than others [2,6,32].

To determine if there was a link between levels or kinetics of CWSS induction and the importance of the CWSS for corresponding resistance phenotypes, we determined the MICs of BB255 compared to BB255ΔVraR for all of the antibiotics tested above and calculated the fold reduction in MIC (Table 2). BB255ΔVraR contains a non-polar deletion truncating VraR after the 2^nd ^amino acid, while leaving the autoregulatory operon intact. The impact of VraR inactivation on resistance phenotypes was very similar to those previously published for deletion of *vraSR *in *S. aureus *N315 [2]. The majority of MICs decreased in the VraR mutant compared to the parent strain BB255 (Table 2). The largest impact seen was on the flavomycin MIC, which decreased 16-fold. Bacitracin and teicoplanin MICs were also much lower, with both reduced by 10-fold, and were similar to values previously published for *vraSR *null-mutants [2]. In contrast to Pietiänen et al. [32], who saw no effects on the vancomycin MIC in a *vraSR *deletion mutant of strain Newman, we observed a 2-fold decrease in vancomycin MIC, similar to that observed by Kuroda et al. in strain N315 [2]. Our results, which showed a weak 2-fold reduction in fosfomycin MIC and no impact on D-cycloserine resistance, also agreed with those obtained for the N315 *vraSR *deletion mutant. While previous reports gave conflicting results concerning the effect of VraSR inactivation on daptomycin resistance [9,32], we observed a reproducible 2-fold reduction in MIC upon VraR inactivation, supporting results from Muthaiyan et al. [9].

Inactivation of VraR had no effect on oxacillin resistance in the methicillin susceptible *S. aureus *(MSSA) strain BB255. However, inactivation of *vraR *in BB270, an MRSA isogenic to BB255 that contains a type I *SCCmec*, reduced the oxacillin MIC from >256 to 64 μg/ml [26], to similar levels as those reported for other *vraSR *mutants in MRSA strains [2,6,33]. Loss of VraR also rendered the mutant 2-fold more susceptible to the action of lysostaphin and 4-fold more susceptible to tunicamycin; phenotypes which have not been previously published for VraSR mutants.

These results confirmed that the ability to induce the cell wall stress stimulon confers varying levels of protection against the effects of cell wall active agents. However, comparison of our MIC results with our induction data revealed no clear links between how quickly, or to which maximal level, the antibiotics are able to induce the CWSS and the impact of a functional VraSR signal transduction response on resistance levels to those antibiotics.

The *sas016 *promoter-luciferase fusion construct was also analysed in BB255ΔVraR. Expression levels of p*sas016*_p_-*luc*+ in BB255ΔVraR in uninduced samples were ~10-fold lower than in the wild type BB255. BB255ΔVraR p*sas016*_p_-*luc*+ was induced with 5x MIC of fosfomycin, D-cycloserine, tunicamycin, bacitracin, flavomycin, vancomycin, teicoplanin, oxacillin and daptomycin and 1x MIC of lysostaphin, for 60 min. The luciferase activities ranged from 1.5-fold higher to 10-fold lower than those in uninduced cultures, showing that none of the antibiotics used could induce *sas016 *expression in absence of VraR.

## Conclusions

In this study, we describe the application of a highly sensitive luciferase-reporter gene construct for indirectly measuring CWSS induction kinetics in *S. aureus*. This system was used to compare induction characteristics of ten different cell wall active antibiotics with diverse enzymatic targets or modes of action. Induction of the CWSS by all ten antibiotics could be precisely quantified and while all ten antibiotics induced the CWSS, induction patterns varied greatly and were highly antibiotic-specific. Each antibiotic produced unique induction curves, which differed in lag times before induction, maximal rates of induction and peak induction levels.

Induction kinetics were also strongly antibiotic concentration-dependent, to different extents for each antibiotic, and generally correlated inversely with decreasing OD values, therefore linking induction kinetics to antibiotic activity. However, there were no obvious trends linking antibiotics acting on similar stages of CWSS with specific induction patterns. Therefore, the signal triggered by all of the antibiotics, that is responsible for activating VraS signal transduction, does not appear to be linked to any particular enzymatic target, as CWSS induction was triggered equally strongly by antibiotics targeting early cytoplasmic stages (e.g. fosfomycin) and late extracellular polymerization stages (e.g. oxacillin) of peptidoglycan synthesis. This is a key difference between the VraSR system of *S. aureus *and the homologous LiaRS systems of other Gram-positive bacteria such as *B. subtilis *and *S. mutans*, which are only activated by lipid-II interacting antibiotics, such as bacitracin, ramoplanin and nisin [15-18]. The increased induction spectrum could account for the larger size of the *S. aureus *CWSS and its protective role against more different classes of antibiotics. Although no direct links between induction properties and the impact of the CWSS on respective resistance phenotypes could be found.

Previous studies have reported large differences in CWSS induction characteristics. However, most studies were performed on different strains and using different experimental conditions. Variations in characteristics observed for the ten antibiotics tested here, indicated that each antibiotic has optimal induction conditions that should be determined before CWSS studies are carried out, including the right antibiotic concentration for the strain used and the optimal sampling time point to measure maximal induction.

## Authors' contributions

VD carried out most of the experimental work and drafted the manuscript. PS and BB participated in the design and coordination of the study and helped to draft the manuscript. RH participated in the microbiological studies and helped to draft the manuscript. NM participated in the design and coordination of the study, carried out molecular biological studies and helped to draft the manuscript. All authors read and approved the final manuscript.
